# Body mass index, physical activity and dental caries: cross-sectional HUNT4 oral health study

**DOI:** 10.1038/s41598-025-12282-2

**Published:** 2025-08-04

**Authors:** Lin Jiang, Neda Kordy, Arnhild Myhr, Lina Stangvaltaite-Mouhat, Rasa Skudutyte-Rysstad, Laxmi Bhatta, Abhijit Sen

**Affiliations:** 1 Center for Oral Health Services and Research (TkMidt), Trondheim, Norway; 2 Department of Public Health and Nursing, Faculty of Medicine and Health Science, Norwegian University of Science and Technology, Trondheim, Norway; 3 Oral Health Centre of Expertise in Eastern Norway, Oslo, Norway; 4 HUNT Center for Molecular and Clinical Epidemiology, Department of Public Health and Nursing, Faculty of Medicine and Health Science, Norwegian University of Science and Technology, Trondheim, Norway; 5 FIU-PH, Division of Mental Health Care, St Olav’s Hospital, Trondheim, Norway; 6 Department of Clinical and Molecular Medicine, Norwegian University of Science and Technology, Trondheim, Norway; 7 Department of Mental Health, Faculty of Medicine and Health Science, Norwegian University of Science and Technology, Trondheim, Norway

**Keywords:** BMI, Physical activity, Dental caries, Oral health, The HUNT4 oral health study, Diseases, Risk factors

## Abstract

Studies on the association between body mass index (BMI) and dental caries among adults are limited. Moreover, individuals with a high BMI may be either physically active or inactive, but the impact of these combinations on dental caries remains unexplored. In this study, we aimed to investigate the associations between BMI, its combination with physical activity (PA), and dental caries in the adult population. We conducted a cross-sectional analysis using data from Norwegian HUNT4 Oral Health Survey (2017–2019). BMI was categorized as < 25.0 (normal), 25.0–29.9 (overweight), or ≥ 30.0 kg/m^2^ (obese). The combination of BMI and PA was classified into 4 groups: (1) normal weight and active; (2) normal weight and inactive; (3) overweight-obese and active; and (4) overweight-obese and inactive. Main outcomes included the total number of decayed, missing, and filled teeth (D_3_MFT) and decayed teeth (D_3_T), while missing and sound teeth were secondary outcomes. Ratios of means (RM) with 95% confidence intervals (CI) were calculated using negative binomial regression. Effect modification by age (< 65 vs. ≥ 65 years) was assessed via the likelihood ratio test. We included 4752 individuals with a mean age of 51.9 years (SD 15.9). Compared to individuals with BMI < 25 kg/m^2^, those with BMI ≥ 30.0 kg/m^2^ was associated with an increased mean number of D_3_MFT (adjusted RM: 1.10, 95% CI 1.07–1.13), D_3_T (1.19, 95% CI 1.07–1.32), and missing teeth (1.11, 95% CI 1.00–1.22), but inversely with sound teeth (0.96, 95% CI 0.92–0.99). No combined effect of BMI and PA was observed. The association between BMI and dental caries was modified by age, with an association observed in individuals under 65 years (*P*_*likelihood ratio test*_ < 0.001). We observed that a higher BMI was associated with a higher dental caries experience and missing teeth. However, there was no evidence of a combined effect between BMI and PA on dental caries.

## Introduction

Dental caries is the most common non-communicable disease globally^1,2^. Nearly 2.3 billion individuals were reported to have untreated caries in the permanent dentition according to a recent Global Burden of Disease Study^3^. Although oral health has improved over the last four decades in Norway, dental caries is still prevalent among adults^4^. It is a leading cause of tooth loss, significantly diminishing quality of life and posing a considerable economic and public health challenge^5^.

Obesity, as measured by body mass index (BMI), is often linked to high sugar consumption and lower socioeconomic status, both of which are also important risk factors for the development of dental caries^6–8^. On the other hand, obesity has been reported to be associated with chronic inflammation, which may impact oral health among other diseases, including dental caries^9^. Moreover, individuals with obesity may experience changes in salivary flow or composition, potentially increasing their susceptibility to dental caries^10^, suggesting a potential link between obesity and dental caries.

Few studies have examined the associations between BMI and dental caries in adult populations^11–15^, and those have generally reported inconsistent findings. Some studies have found a positive association^11^ while others have reported no^12,13^, inverse association^14^ or U-shaped pattern^15^. Given the limited number of studies examining this association in adults, we believe that further research is needed.

In addition to BMI, research suggests that physical activity (PA) may also influence oral health^16^. Individuals with high BMI can vary widely in their physical activity levels; some may remain active despite excess weight, while others are largely inactive. Those who are both overweight/obese and physically inactive tend to exhibit most sedentary behaviors and related metabolic risks compared to those with only one of these risk factors^17–19^. Sedentary behavior has been associated with metabolic dysregulation, systemic inflammation, and immune dysfunction^20–22^, all of which may contribute to increased susceptibility to oral diseases, including dental caries.

Given that both obesity and physical inactivity are independently associated with caries related mechanisms such as poor dietary patterns, chronic inflammation, and reduced salivary function, we hypothesize that their combination may have a synergistic effect on the prevalence of dental caries. In this context, a synergistic effect implies that the co-occurrence of high BMI and low PA is associated with a higher prevalence of caries than would be expected from the sum of their individual effects. However, to our knowledge, no previous studies have examined the combined effect of BMI and PA on dental caries in the adult population.

Therefore, this study aimed to investigate the cross-sectional associations between BMI and dental caries in a general Norwegian adult population. Further, we explored the combined effect of BMI and PA on dental caries prevalence and assessed whether these associations were modified by age.

## Methods

### Study population

The HUNT Study is a large, population-based health study that has been conducted in four phases: HUNT1 (1984–1986), HUNT2 (1995–1997), HUNT3 (2006–2008), and HUNT4 (2017–2019) in Trøndelag County, Norway^23,24^. All adults aged 20 years or older were invited to complete general self-reported questionnaires on socio-demographics, lifestyle factors, clinical conditions/diseases, and underwent clinical examinations^23,24^.

**Fig. 1:**
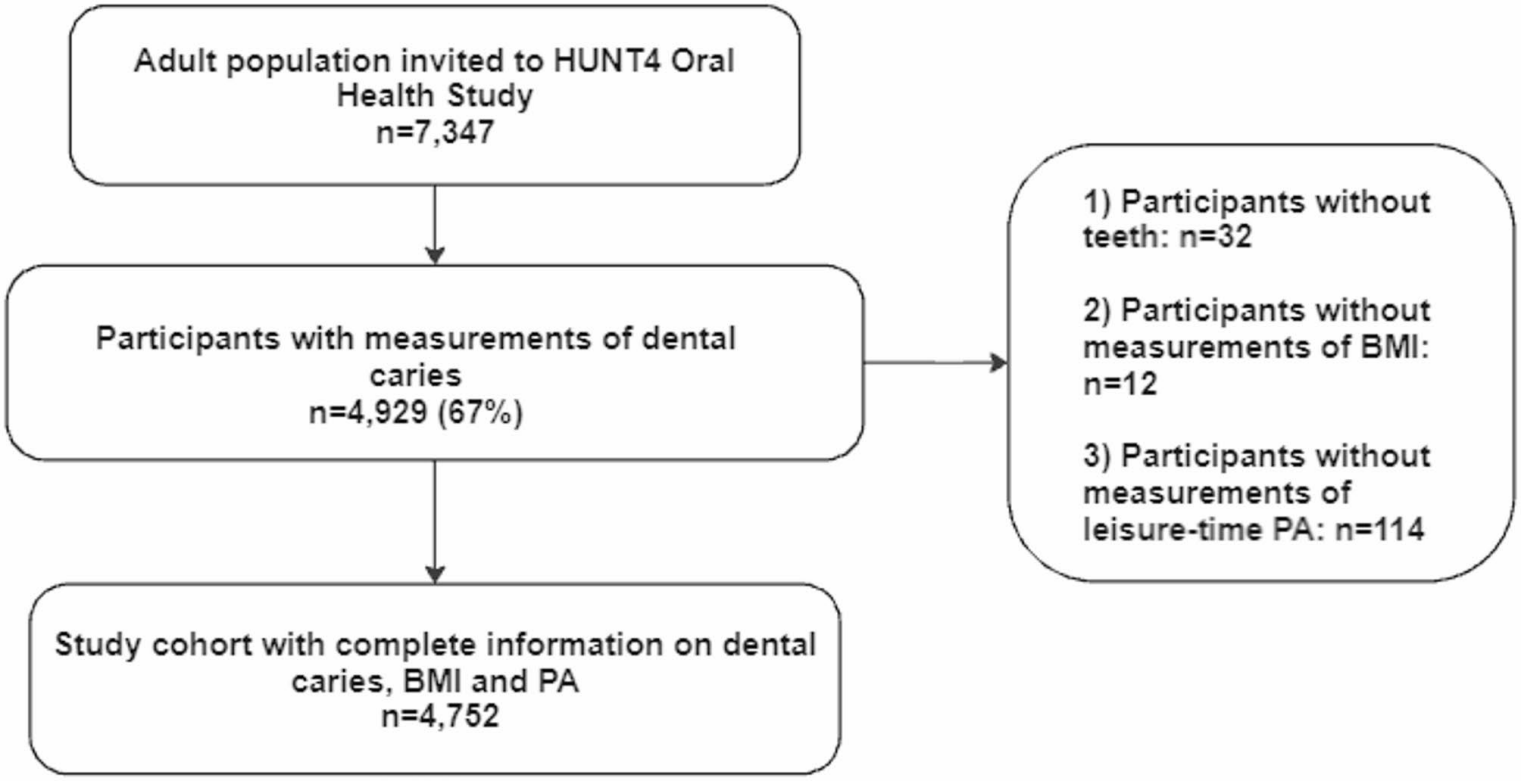
Flow chart of the study population.

A sub-study on oral health was conducted as part of the HUNT4 survey. A random sample of 7347 adults from HUNT4 (20% of the population) was invited to participate in the HUNT4 Oral Health Study^4,23^. Participants in the HUNT4 Oral Health study were aged ≥ 20 years. Among them, 4929 adults underwent clinical and radiographic examinations for dental caries (Fig. 1). Edentulous individuals were excluded (*n* = 32). Additionally, individuals with missing information on study exposures, such as BMI (*n* = 12) and PA (*n* = 114), were excluded, resulting in a final study population of 4752 adults. This sample provides a representative subset of the general adult population in Trøndelag County.

### Exposures

#### Measurements of BMI

In all the HUNT surveys, body weight and height were measured by health professionals at the time of clinical examination. Height was measured to the nearest centimeters, and weight was measured to the nearest 0.5 kg. BMI was calculated as weight in kilograms divided by height squared in meters (kg/m^2^). Following the WHO recommendation, BMI was further categorized into underweight (< 18.5 kg/m^2^), normal weight (18.5–24.9 kg/m^2^), overweight (25–29.9 kg/m^2^), and obese (≥ 30 kg/m^2^)^25^. Due to the small number of underweight individuals (*n* = 36), the underweight and normal weight categories were combined into a single group.

#### Combination of BMI and PA.

In all the HUNT surveys, PA was self-reported average leisure-time PA based on questions about frequency, intensity, and duration: Question (1) “*How frequently do you exercise?*” with response scores of ‘never’ (0), ‘less than once a week’ (0.5), ‘once a week’^1^, ‘two to three times per week’ (2.5), and ‘almost every day’^5^. Question (2) “*If you do such exercise as frequently as once or more times a week*,* how hard do you push yourself ?*” with response scores of ‘I take it easy, I don’t get out of breath or break a sweat^1^, ‘I push myself until I’m out of breath and break into a sweat’^2^ and ‘I practically exhaust myself’^3^ and Question (3) “*How long does each session last?*’ with response scores of ‘less than 15 min’ (0.1), ‘15–29 min’ (0.38), ‘30 min to 1 h’ (0.75), and ‘more than 1 h’ (1.0). We calculated a PA summary score (PAS) by multiplying each participant’s score on the three questions. We chose to categorize physical activity based on the PAS, following the approach used in the previous HUNT study. This PAS was suggested to be reliable and valid^26^. The individuals who responded as never or less than once a week for the frequency of PA were those with no PA (PAS = 0). The remaining individuals were categorized into low PA (0 < PAS ≤ 1.90), medium PA (1.91 ≤ PAS ≤ 3.75), and high PA (3.76 ≤ PAS ≤ 15.00) based on tertile values of the PAS^27^. The individuals with no and low PA were further regarded as physically inactive, whereas individuals with moderate to high PA were regarded as active. We further classified the combination of BMI and PA into four groups: a) normal weight and physically active; b) normal weight and physically inactive; c) overweight/obese and physically active; and d) overweight/obese and physically inactive.

### Outcome

Information on dental caries was extracted from The HUNT4 Oral Health Study. In brief, trained and calibrated dentists or dental hygienists registered all individuals’ caries experience via both clinical and radiographic bitewing examinations. Cohen’s kappa values for inter-examiner agreement during the clinical examinations ranged from 0.71 to 0.91 across the different field stations, indicating substantial to almost perfect agreement. Additionally, repeated evaluations of a standardized set of clinical intra-oral photographs were conducted at the beginning (kappa = 0.82) and later in the data collection period (kappa = 0.85) to assess inter-examiner reliability. Intra-examiner agreement, based on the same set of photographs, yielded kappa values ranging from 0.79 to 0.93. For radiographic caries assessment, inter-examiner reliability showed kappa values of 0.87 and 0.84 in the first and second calibration sessions, respectively, while intra-examiner reliability ranged from 0.84 to 0.87. A more detailed description for training and calibration of examiners has been published previously^4^.

Dental Caries was classified using the system developed by Amarante (1998)^28^, which is the standard diagnostic classification taught in all dental schools in Norway and commonly used in clinical practice. This system grades carious lesions on a scale from 1 to 5, based on visual and radiographic criteria. Grade 1 indicates discoloration without substance loss and no radiographic findings, while Grade 2 involves minimal enamel breakdown or caries confined to the enamel. In this study, we included only dentine caries, defined as Grades 3 to 5: lesions with moderate to severe substance loss and radiographic evidence of caries extending into the outer, middle, or inner third of the dentin. This corresponds to the WHO D_3_ threshold for cavitated dentine lesions. The total dental caries experience, number of decayed, missing, and filled teeth (D_3_MFT), was regarded as the main outcome. Number of teeth with untreated dentine caries (D_3_T) were defined as teeth with primary or secondary caries in dentine (caries registration grades 3–5) and root caries with cavitation^4^, based on clinical examination and bitewing radiographs^4,28^ and regarded as another main outcome. The missing teeth (MT) referred to all missing teeth regardless of the reason^4^. The filled teeth (FT) were restored teeth without secondary caries and include all types of filling materials and crowns^4^. In addition, the number of sound teeth (ST) were calculated and characterized by the absence of initial or dentine caries, filling and/or other restorations or bridge abutments^28^. Both MT and ST were used as secondary outcomes.

### Covariates

All potential covariates in this study were collected via questionnaires at HUNT4. Age in years (continuous), sex (females, males), smoking status (never, former, and current), sugary drink intake (almost not, 1–6 glasses per week, and ≥ 2 per day).

Education and income level were categorized consistently with previous HUNT studies^29,30^. Education level, originally grouped in six categories from the questionnaire, was condensed into three groups (≤ 10 years, 11–14, and > 14 years) were included. Household years’ income before taxation, recorded in Norwegian Kroner (NOK), originally grouped into five categories in the questionnaire, were condensed into three groups (< 450,000 kr, 451,000-1000,000 kr and > 1000,000 kr). We used the original HUNT4 questionnaire categories for fruit intake and vegetable intake, each defined separately as: less than once, 1–3 times per week, 4–6 times per week, and daily. Based on these, we constructed a combined variable for ‘fruit or vegetable intake’ with three categories: ≤ 3 times per week, 4–6 times per week, and daily. Similarly, snus use ‘moist snuff’ was originally categorized as never, former, and current, but was recoded into two categories: ever users vs. never users. Diabetes status was assessed by asking, ‘Have you ever had, or do you currently have diabetes?’ with response options: Yes or No. Missing data for each variable were included in the analyses as an “unknown” category.

### Statistical analysis

Descriptive statistics of the study individuals were provided, with means (SD) for continuous variables and frequencies (percentages) for categorical variables. The characteristics of the individuals were presented by BMI categories and by combinations of BMI and PA levels. We assessed the association between BMI levels, their combination with PA, and dental caries using negative binomial regression. To study the combined effect of BMI and PA on dental caries, four combined categories were constructed (normal weight and physically active as *reference group*, normal weight and physically inactive, overweight/obese and physically active, overweight/obese and physically inactive).

The ratio of means (RM) with 95% confidence intervals (CIs) was used to evaluate the associations. We employed negative binomial regression due to the count nature of the outcome variables and the potential for overdispersion^31,32^. In a cross-sectional setting, the mean number is increasing when RM > 1 while the mean number is decreasing when RM < 1^32^. While assessing the association between exposure and outcome, the confounders were selected based on previous knowledge^11–15^. The adjusted model for the association between BMI and dental caries included the following confounders: age, sex, smoking status, sugary drink intake, PA, education level, and income level. We further examined the potential effect modification by age (< 65 vs. ≥ 65y) using the likelihood ratio test. Additionally, an alternative cut-off at age 55y was used to distinguish between the pre-fluoride and fluoride generations, reflecting differences in fluoride exposure during formative years, which may influence long-term oral health outcomes such as dental caries.

To test the robustness of the results, we: (i) further adjusted for additional confounders, including snus consumption (no, yes), fruit or vegetable intake (≤ 3 times per week, 4–6 times per week, and daily) and ever diabetes status (yes, no) for all the associations; (ii) used MT and ST as secondary outcomes; and (iii) redefined the combination groups into normal weight and active, normal weight and non-active, overweight/obese and non-active, and overweight/obese and active. All the statistical analyses were performed with STATA/SE 16.1 (College Station, TX, USA).

## Results

A total of 4752 individuals with a mean age of 51.9 years (SD 15.9) were included in the study. Compared to individuals with a BMI < 25 Kg/m^2^, those with higher BMI (≥ 30.0 kg/m^2^, obese) were more likely to be smokers, less physically active, and less educated (Table 1).

**Table 1:** Characteristics of participants according to body mass index levels in the HUNT4 oral health study (*n* = 4752).

	BMI (kg/m^2^)		
	< 25.0	25.0─29.9 (Overweight)	≥ 30.0 (Obese)
N	1696	1988	1068
D_3_MFT	13.2 ± 7.2	15.7 ± 6.7	15.9 ± 6.4
D_3_T	1.2 ± 1.7	1.4 ± 1.9	1.5 ± 2.0
Age (Mean ± SD)	47.8 ± 17.6	53.9 ± 15.4	52.8 ± 14.8
Age (years),%			
< 65	80.1	73.3	77.2
≥ 65	19.9	26.7	22.8
Female, %	64.3	49.0	53.8
Smoking status, %			
Never	52.5	42.9	40.3
Former	39.2	49.1	51.0
Current	8.0	7.7	8.5
Unknown	0.3	0.3	0.2
Sugary drink intake (glass), %			
Almost not	56.1	57.1	57.4
1–6 per week	38.4	38.3	36.1
≥ 2 per day	4.0	3.0	4.7
Unknown	1.4	1.6	1.8
Leisure-time physical activity^a^, %			
No	10.6	15.9	20.7
Low	25.5	28.9	30.9
Medium	29.1	27.8	29.5
High	34.8	27.3	18.9
Education (years), %			
≤ 10	5.5	7.5	7.5
11─14	42.7	49.0	51.0
> 14	51.7	43.2	41.0
Unknown	0.2	0.2	0.5
Household income^b^ (NOK), %			
Low (< 450.000)	25.8	23.3	26.7
Middle (451.000─1.000.000)	47.6	54.8	52.9
High (> 1.000.000)	24.2	20.1	19.3
Unknown	2.4	1.8	1.1

BMI, Body mass index; D_3_MFT, The Decayed, Missing and Filled Teeth index; D_3_T, Decayed dentine teeth; HUNT, The Trøndelag Health Study. MT, Missing teeth regardless of the reason; ST, Teeth without initial or dentine caries, filling and/or other restorations and without bridge abutment. NOK, Norwegian Kroner. ^a^Leisure-time physical activity was self-reported and a summary score of frequency, duration and intensity was calculated to represent no (0.00), low (> 0.00, ≤ 1.90), moderate (1.91–3.75) and high (3.76–15.00) leisure-time physical activity. ^b^Household Income (low:< 450,000 kr, middle: 451,000–1000,000 kr, high: >1000,000 kr, unknown).

The individuals who were overweight/obese and inactive seemed to be the oldest, had the highest proportion of former smokers, and generally had less education and low-income level (Table 2).

**Table 2:** Characteristics of participants according to different combinations of body mass index and leisure-time physical activity levels in the HUNT4 oral health study (*n* = 4752).

	Normal weight & active	Combination of BMI and leisure-time PA^a^		
		Normal weight & inactive	Overweight/obese& active	Overweight/obese & inactive
N	1084	612	1613	1443
D_3_MFT	13.2 ± 7.1	13.3 ± 7.3	15.7 ± 6.4	15.9 ± 6.7
D_3_T	1.1 ± 1.6	1.4 ± 1.9	1.4 ± 1.8	1.5 ± 2.0
Age (Mean ± SD)	48.1 ± 17.5	47.2 ± 17.9	53.3 ± 14.7	53.8 ± 15.9
Age (years), %				
< 65	80.4	79.7	76.8	72.3
≥ 65	19.6	20.3	23.2	27.7
Female, %	65.3	62.6	51.5	49.8
Smoking status, %				
Never	54.7	48.5	45.9	37.6
Former	39.5	38.7	49.2	50.5
Current	5.4	12.7	4.8	11.5
Unknown	0.5	0.0	0.1	0.4
Sugary drink intake (glass), %				
Almost not	61.6	46.4	61.6	51.2
1–6 per week	34.9	44.8	33.6	41.9
≥ 2 per day	1.8	8.0	2.4	4.9
Unknown	1.7	1.0	1.4	2.0
Education (years), %				
≤ 10	4.3	7.5	5.4	9.9
11─14	39.3	48.7	48.2	51.4
> 14	56.3	43.5	46.1	38.3
Unknown	0.1	0.3	0.2	0.3
Household income (NOK) ^b^, %				
Low	22.6	31.5	20.6	28.8
Middle	48.8	45.6	55.2	52.9
High	26.6	19.9	22.7	16.6
Unknown	2.0	2.9	1.5	1.7

BMI, Body mass index; D_*3*_MFT, The Decayed, Missing and Filled Teeth index; D_3_T, Decayed dentine teeth; HUNT, The Trøndelag Health Study; MT, Missing teeth regardless of the reason; PA, physical activity; ST, Teeth without initial or dentine caries, filling and/or other restorations and without bridge abutment.^a^Participants were categorized into four groups according to different combinations of BMI and leisure-time PA: 1) normal weight & active; 2 )normal weight & inactive; 3)overweight/obese & active;4) overweight/obese & inactive. Due to limited cases in underweight group (36 cases), underweight and normal weight were collapsed into one group as normal weight. Participants who were inactive were those with no or low PA. Active participants were those with moderate or high PA. ^b^ NOK: Norwegian Kroner. Income (low:<450,000 kr, middle: 451,000-1000,000 kr, high: >1000,000 kr, unknown).

We observed positive associations between being overweight (RM, 1.19, 95% CI 1.15–1.23) and obese (RM, 1.20, 95% CI 1.16–1.24) with D_3_MFT, compared to individuals with a BMI < 25 kg/m^2^ in an unadjusted model (Table 3). After adjusting for confounders, the associations attenuated to an RM of 1.05 (95% CI 1.03–1.08) for overweight and 1.10 (95% CI 1.07–1.13) for obese individuals. The positive associations between BMI and D_3_T were slightly stronger than those for D_3_MFT, with RM of 1.15, 95% CI 1.05–1.26 for overweight, and 1.19, 95% CI 1.07–1.32 for the obese group in the adjusted model. Further, individuals with obesity had a higher mean number of MT, but a lower mean number of ST compared to individuals with normal weight.

**Table 3:** Cross-sectional association between body mass index and dental caries (D_3_MFT, D_3_T), missing teeth (MT), and sound teeth (ST) in the HUNT4 oral health study (*n* = 4752) using negative binomial regression models.

Outcome	BMI (Kg/m^2^)	*N*	Unadjusted model		Adjusted model^a^	
			RM	95% CI	RM	95% CI
D_3_MFT	Normal^b^ (< 25.0)	1696	1.00	Reference	1.00	Reference
	Overweight (25.0-29.9)	1988	1.19	1.15–1.23	1.05	1.03–1.08
	Obese (≥ 30.0)	1068	1.20	1.16–1.24	1.10	1.07–1.13
D_3_T	Normal^b^ (< 25.0)	1696	1.00	Reference	1.00	Reference
	Overweight (25.0–29.9)	1988	1.18	1.08–1.29	1.15	1.05–1.26
	Obese (≥ 30.0)	1068	1.25	1.13–1.39	1.19	1.07–1.32
MT	Normal^b^ (< 25.0)	1696	1.00	Reference	1.00	Reference
	Overweight (25.0–29.9)	1988	1.31	1.18–1.46	0.99	0.91–1.09
	Obese (≥ 30.0)	1068	1.45	1.29–1.64	1.11	1.00–1.22
ST	Normal^b^ (< 25.0)	1696	1.00	Reference	1.00	Reference
	Overweight (25.0–29.9)	1988	0.83	0.80–0.86	0.99	0.96–1.02
	Obese (≥ 30.0)	1068	0.81	0.77–0.84	0.96	0.92–0.99

BMI, Body mass index; CI Confidence Interval; D_3_MFT, Numbers of Decayed, Missing and Filled teeth; D_3_T, Decayed dentine teeth; HUNT, The Trøndelag Health Study; MT, Missing teeth regardless of the reason; RM, Ratio of means; ST, Teeth without initial or dentine caries, filling and/or other restorations and without bridge abutment. ^a^Adjusted for age (continuous in years), sex(male/female), smoking status (never/former/current/unknown), sugary drink intake (almost not/ 1–6 glass per week/≥2 per day/unknown), leisure-time physical activity (no/low/moderate/high), education (≤ 10 years/11–14 years/>14/unknown) and income (< 450,000 kr /451,000-–1000,000 kr />1000,000 kr /unknown). ^b^Normal and underweight categories were collapsed into one group since there were only 36 underweight participants.

Compared to individuals who were of normal weight and physically active, those who were of normal weight and physically inactive showed no difference in the mean number of D_3_MFT in the adjusted model (Table 4). However, similar higher RMs were observed for individuals who were overweight/obese and physically active (RM: 1.08, 95% CI 1.05–1.11) and overweight/obese and physically inactive (RM: 1.06, 95% CI 1.03–1.10).

Furthermore, compared to individuals who were of normal weight and physically active, those who were normal weight and physically inactive showed higher RM of D_3_T in the adjusted model. Among the groups, individuals who were overweight/obese and inactive appeared to have the highest RM of D_3_T. Additionally, individuals who were of normal weight and physically inactive had the highest RM for MT, while those who were overweight/obese and physically inactive had the lowest RM for ST.

**Table 4:** Cross-sectional association between combination of body mass index and leisure-time physical activity and dental caries (D_3_MFT, D_3_T), missing teeth (MT) and sound teeth (ST) in the HUNT4 oral health study (*n* = 4752) using negative binomial regression models.

Outcome	Combination of BMI and PA	*N*	Unadjusted model		Adjusted model^a^	
			RM	95% CI	RM	95% CI
D_3_MFT	Normal weight & active^b^	1084	1.00	Reference	1.00	Reference
	Normal weight & inactive^c^	612	1.01	0.96─1.07	1.01	0.97─1.05
	Overweight/obese & active^b^	1613	1.19	1.14─1.24	1.08	1.05─1.11
	Overweight/obese & inactive^c^	1443	1.21	1.16─1.25	1.06	1.03─1.10
D_3_T	Normal weight & active^b^	1084	1.00	Reference	1.00	Reference
	Normal weight & inactive^c^	612	1.26	1.09─1.44	1.17	1.02─1.34
	Overweight/obese & active^b^	1613	1.25	1.12─1.39	1.22	1.09─1.35
	Overweight/obese & inactive^c^	1443	1.40	1.25─1.56	1.29	1.16─1.44
MT	Normal weight & active^b^	1084	1.00	Reference	1.00	Reference
	Normal weight & inactive^c^	612	1.37	1.16─1.62	1.24	1.07─1.44
	Overweight/obese & active^b^	1613	1.37	1.20─1.56	1.12	1.01─1.24
	Overweight/obese & inactive^c^	1443	1.73	1.52─1.97	1.17	1.05─1.30
ST	Normal weight & active^b^	1084	1.00	Reference	1.00	Reference
	Normal weight & inactive^c^	612	0.98	0.92─1.03	0.99	0.95─1.03
	Overweight/obese & active^b^	1613	0.83	0.80─0.87	0.98	0.95─1.01
	Overweight/obese & inactive^c^	1443	0.80	0.76─0.83	0.97	0.93─1.00

BMI, Body mass index; CI Confidence Interval; D_3_MFT, Numbers of Decayed, Missing and Filled teeth; D_3 − 5_T, Decayed dentine teeth; HUNT, The Trøndelag Health Study; MT, Missing teeth regardless of the reason; PA, physical activity; RM, Ratio of means; ST, Teeth without initial or dentine caries, filling and/or other restorations and without bridge abutment. ^a^Adjusted for age (continuous in years), sex(male/female), smoking status (never/former/current/unknown), sugary drink intake (almost not/ 1–6 glass per week/≥2 per day/unknown), education (≤ 10 years/11–14 years/>14/unknown) and income (< 450,000 kr /451,000-1000,000 kr />1000,000 kr /unknown) ^b^Active referred to medium to high PA.^c^Inactive referred to no and low PA.

There was statistical evidence of effect modification by age (*P*_likelihood ratio test_ <0.001). A stronger association between BMI and D_3_MFT was observed only among individuals younger than 65 years, while no associations were found among those ≥ 65 years (Table 5). Similar results were observed when D_3_T was used as the outcome (see Supplementary Table 3. Notably, the results did not change when we used an alternative cut-off at age 55-year threshold, suggesting that the observed trends were robust across both age definitions.

**Table 5:** Cross-sectional association between body mass index, its combination with physical activity and dental caries experience (D_3_MFT) in the HUNT4 oral health study, stratified by age (*n* = 4752) using negative binomial regression models.

		Exposure		Adjusted Model^1^	
Outcome	Age (years)	BMI (Kg/m^2^)	*N*	RM	95% CI
D_3_MFT	< 65	Normal^2^ (< 25.0)	1359	1.00	Reference
		Overweight (25.0-29.9)	1458	1.19	1.14─1.23
		Obese (≥ 30.0)	825	1.21	1.16─1.27
	≥ 65	Normal^2^ (< 25.0)	337	1.00	Reference
		Overweight (25.0-29.9)	530	1.00	0.97─1.02
		Obese (≥ 30.0)	243	0.99	0.96─1.01
		**Combination of BMI and PA**			
D_3_MFT	< 65	Normal weight & active^3^	871	1.00	Reference
		Normal weight & inactive^4^	488	0.99	0.93─1.06
		Overweight/obese & active^3^	1239	1.21	1.15─1.26
		Overweight/obese & inactive^4^	1044	1.18	1.12─1.24
	≥ 65	Normal weight & active^3^	213	1.00	Reference
		Normal weight & inactive^4^	124	1.00	0.97─1.04
		Overweight/obese & active^3^	374	0.99	0.96─1.02
		Overweight/obese & inactive^d^	399	1.00	0.97─1.03

BMI, Body mass index; CI Confidence Interval; D_3_MFT, Numbers of Decayed, Missing and Filled teeth; HUNT, The Trøndelag Health Study; RM, Ratio of means. ^a^Adjusted for sex(male/female), smoking status (never/former/current/unknown), sugary drink intake (almost not/ 1–6 glass per week/≥2 per day/unknow), education (≤ 10 years/11–14 years/>14/unknown) and income (< 450,000 kr /451,000-1000,000 kr />1000,000 kr /unknown). Additional adjustment for leisure-time physical activity (no/low/moderate/high) for the association between BMI and D_3_MFT. ^b^Normal and underweight categories were collapsed into one group since there were only 36 underweight participants; ^c^Active referred to medium to high PA. ^d^Inactive referred to no and low PA.

When we performed sensitivity analysis with additional adjustments for confounders such as snus users, fruit or vegetable intake, and diabetes status, the results remained consistent with those of our main analyses. Furthermore, when we redefined the combined groups, classifying no PA as inactive and any PA from low to high as active, the results remained similar for D_3_MFT, D_3_T, MT, and ST (Supplementary Table S1).

## Discussion

In this population-based cross-sectional study of Norwegian adults, we observed a positive association between BMI and dental caries experience, especially pronounced for untreated caries. However, no combined effect of BMI and PA on dental caries experience was observed. Additionally, age seemed to modify the association, with positive associations only observed in individuals less than 65 years old.

In line with our findings, a cross-sectional study from Australia (including 3,745 individuals aged 15–91 years) suggested a positive association between BMI and D_3_MFT^11^. Another study from Israel, including 66,790 adults aged between 18 and 50 years, suggests that both underweight and overweight/obese to be associated with a higher prevalence of D_3_T, suggesting a non-linearly U-shaped pattern^15^. In contrast, an inverse association was observed in a cross-sectional study conducted by Song et al., including 16,129 Korean adults aged 19 years and older^14^. Another cross-sectional study using data from 8144 U.S. adults aged over 20 years from the pre-pandemic cycle of the National Health and Nutrition Examination Survey (NHANES) found no association between BMI and dental caries^13^ This discrepancies in findings suggests that the association relationship between BMI and dental caries in adults is complex and may be influenced by factors such as dietary habits, healthcare access, cultural practices, or genetic predispositions in different populations. Notably, it is important to account for sugar consumption when examining the association between BMI and dental caries, as it plays a pivotal role in both obesity and caries development^11^. However, the study by Song et al. did not consider sugar consumption, potentially introducing confounding bias^14^, which may explain the finding of an inverse association. In our study, we accounted for key confounders, including sugary drink intake, ensuring a more accurate analysis. Additionally, we employed detailed and reliable measures of dental caries, capturing both total dental caries experience and untreated dentine caries. To further validate our findings, we used MT and ST as secondary outcomes. Consistent with our findings, studies by Hilgert et al.^33^ and Prpić et al.^34^ also reported that a greater number of MT was associated with higher BMI. The consistency across various dental caries outcomes underscores the significant role of BMI in dental caries development.

Notably, we observed a stronger positive association between BMI and dental caries in individuals younger than 65 years, whereas no association was found in elderly individuals. This difference may be attributed to age-related metabolic and physiological changes^35,36^. Evidence suggests that obesity is more strongly linked to metabolic conditions, such as insulin resistance and systemic inflammation, in younger adults compared to elderly individuals^36^. These metabolic conditions might alter immune responses and susceptibility to infections, including dental caries. On the other hand, the possibility of a reverse relationship, where high caries experience may affect metabolic conditions through altered nutritional and dietary patterns, cannot be ruled out. Furthermore, age-related physiological changes, such as shifts in salivary composition and flow rate, may primarily explain the observed variation in the association between BMI and dental caries by age^37^. Furthermore, **no** combined effect of BMI and PA on dental caries experience was found in our study. Specifically, the most sedentary group, those who were overweight or obese and physically inactive, did not exhibit the highest RM of D_3_MFT and MT. However, PA was inversely associated with untreated caries (Supplementary Table S2), a finding consistent with some previous observational studies^16,38,39^.

Physical activity has been associated with healthier lifestyle behaviors, such as improved dietary habits and better oral hygiene practices, which may contribute to a lower number of decayed teeth^16,39^. However, our study did not observe a direct association between PA and D_3_MFT, indicating that while PA may influence caries development, other factors, such as dietary sugar intake, oral hygiene practices, and access to dental care, likely play significant roles. This underscores the multifactorial nature of dental caries and the need for further research to explore these interactions.

Our study has several strengths. First, the HUNT Study is one of the largest population health-based studies in Norway. By focusing on a general Western population, its findings are more directly comparable to similar populations, enhancing the generalisability of the results. Second, dental caries experience was evaluated based on detailed clinical and radiographic examinations. In addition, we assessed MT and ST as secondary outcomes. These reduce misclassification of the outcome variables and provide a more accurate assessment of the dental caries burden. Third, we adjusted for important confounders, including lifestyle factors (smoking), socioeconomic indicators (education and income), and weekly sugary drink intake. This allowed for more robust control of confounding bias compared to the previous studies^14,15^.

This study has several limitations. First, since this is a cross-sectional study, we cannot establish a temporal association between exposure and the outcome. Second, selection bias may exist since participating individuals were generally healthier than non-participating individuals in the HUNT Study^24^. In addition, the HUNT4 Oral Health Study reported an overrepresentation of individuals with higher education^4^, which may have led to an underestimation of the mean number of teeth with dental caries in the study. Since this underestimation was likely nondifferential, the effect estimates for the association between BMI and dental caries may be attenuated, suggesting that the true strength of the association could be stronger than reported. Third, the variable on PA (frequency, duration, intensity) was self-reported, and misclassification might exist. However, the reliability and validity of the HUNT questionnaire for PA has been investigated, and a good correlation between the questionnaire of PA and object measurement, such as maximum oxygen uptake, was reported^26^.

## Conclusion

BMI was suggested to be positively associated with dental caries. However, no combined effect of BMI and PA was found on dental caries. Further studies with larger sample sizes and longitudinal designs accounting for a broad range of confounders are needed to replicate our findings and explore this association comprehensively.

## Data Availability

“Data from the HUNT Study is available upon request to the HUNT Data Access Committee (hunt@ medisin.ntnu.no) when is used in research projects. The HUNT data access information describes the policy regarding data availability (https://www.ntnu.edu/hunt/data)”.

## Abbreviations

BMI: Body mass index
CI: Confidence interval
D_3−_MFT: Decayed teeth, missing teeth and filled teeth
D_3_T: Decayed teeth
FT: Filled teeth
HUNT: The Trøndelag Health Study
MT: Missing teeth
RM: Ratio of means
PA: Physical activity
PAS: Physical activity summary score
ST: Sound teeth
WHO: World Health Organization
